# Bibliometric analysis of traditional Chinese medicine in cancer treatment via immune system modulation (2015–2025)

**DOI:** 10.3389/fimmu.2025.1581885

**Published:** 2025-05-08

**Authors:** Youfeng Lei, Chunyan Chen

**Affiliations:** Department of Pharmacy, Shanghai Public Health Clinical Center, Fudan University, Shanghai, China

**Keywords:** apoptosis, expression, inflammation, extract, activation, antioxidant

## Abstract

**Objective:**

The application of Traditional Chinese Medicine (TCM) in treating cancer by regulating the immune system has garnered significant attention in the academic community. However, comprehensive quantitative analyses in this field remain limited. This study aims to assess the research progress and key trends over the past decade, providing a framework for future studies.

**Methods:**

A comprehensive literature search was conducted on the application of TCM in treating cancer by regulating the immune system from 2015 to 2025 using the Web of Science database. The search terms mainly included cancer, Traditional Chinese Medicine, immunity and so on. Data were analyzed and visualized using Origin, R software, VOSviewer, and CiteSpace.

**Results:**

A total of 2,459 articles were included in the analysis. The number of related publications has steadily increased since 2015. China leads in publication volume and plays a crucial role in international collaboration. The *Journal of Ethnopharmacology* is the leading journal in this field, publishing a substantial number of highly cited studies. Key research areas include keywords such as “apoptosis,” “expression,” “inflammation,” “extract,” “*in vitro*,” “activation,” “antioxidant,” and “NF-kappa B,” focusing on exploring the role, mechanisms, and efficacy of TCM in modulating immune responses.

**Conclusion:**

Research interest in TCM’s role in treating cancer through immune system regulation continues to grow, underscoring its potential in cancer therapy. Current research primarily focuses on the mechanisms by which TCM treats cancer through the modulation of immune cell functions, inhibition of tumor immune evasion, and regulation of immune-related signaling pathways. It also explores its clinical applications and the potential for enhancing the efficacy of immunotherapy.

## 1 Introduction

Cancer is one of the leading causes of death worldwide and has long been a focal point of medical research (1). In recent years, immunotherapy has emerged as a promising treatment modality, achieving remarkable clinical outcomes by activating the human immune system to fight tumors (2). Technologies such as immune checkpoint inhibitors (e.g., PD-1/PD-L1 inhibitors) (3)and cancer vaccines (4) have been widely adopted in the treatment of various cancers and have demonstrated great clinical potential. However, despite the success of immunotherapy in some patients, many still face challenges, including limited efficacy, drug resistance, and immune-related side effects (5). These issues have prompted the medical community to continue exploring new treatments and adjuvant therapies to optimize the effects of immunotherapy and minimize side effects.

TCM, a medical system with thousands of years of history and extensive clinical experience, has garnered increasing global attention in recent years (6). TCM has shown significant potential in enhancing immune function and reducing immunotherapy-related side effects through its unique approach of holistic regulation and syndrome differentiation. Numerous studies have indicated that TCM can support cancer immunotherapy by regulating the immune system, enhancing immune cell activity, inhibiting tumor immune escape mechanisms, and promoting tumor cell apoptosis (7). Additionally, certain components of Chinese herbal medicines have been found to enhance the efficacy of immune checkpoint inhibitors and alleviate immunotherapy-related adverse reactions (8). For example, herbal medicines such as *Ganoderma lucidum*, *Astragalus membranaceus*, and *Atractylodes macrocephala* have been shown to regulate immune responses, improve immune cell function, and alleviate side effects, such as immune rash and liver damage, during immunotherapy (9, 10).

Moreover, Chinese medicine plays a critical role in regulating the tumor microenvironment. Immune escape in the tumor microenvironment is one of the main challenges facing cancer immunotherapy (11). Chinese medicine can enhance the effects of immunotherapy by improving the tumor microenvironment, inhibiting angiogenesis, and regulating immune cell infiltration (12). Compounds such as tanshinone in *Danshen* and astragalus polysaccharides in *Astragalus membranaceus* not only directly target tumor cells but also promote immunotherapy efficacy by modulating immune responses and enhancing immune system function (9, 13).

Despite increasing interest in this topic, current research is primarily centered on individual herbal components or limited experimental studies, with a lack of comprehensive, systematic evaluations of the field’s development. This has created a gap in understanding the overall research landscape, key contributors, collaboration networks, and emerging research hotspots in the field of TCM-based immunotherapy for cancer. Bibliometric analysis provides a powerful and quantitative approach to address this gap (14). By statistically analyzing a large volume of scientific literature, bibliometrics can uncover knowledge structures and research dynamics in a given field. It enables researchers to identify influential authors, institutions, countries, high-frequency keywords, citation networks, and trending topics, thus helping to map the evolution of research and guide future investigations (15).

Therefore, this study aims to conduct a bibliometric analysis of research on TCM in cancer immunotherapy from 2015 to 2025. Specifically, we will (1) identify publication trends and core contributors in this field, (2) explore major research themes, mechanisms of action, and representative herbal components, and (3) analyze emerging trends and potential research directions. By addressing the existing research gap, this study seeks to provide a systematic overview of the current landscape, support the scientific integration of TCM with modern immunotherapy, and offer theoretical foundations for innovative cancer treatment strategies.

## 2 Materials and methods

### 2.1 Data collection

The data for this study were obtained from the Web of Science Core Collection (WOSCC). The search was conducted on January 15, 2025. A comprehensive search was conducted in the WOSCC database using keywords related to *“Traditional Chinese Medicine,” “Immunotherapy,”* and *“Cancer.”* A total of 12558 articles were retrieved. The search strategy was as follows:

((((TS=(“immune” OR “immunotherapy” OR “immunosuppression” OR “autoimmunity” OR “PD-1” OR “PD-L1” OR “CTLA-4” OR “CAR-T”)) AND TS=(cancer* OR tumor* OR neoplasm OR neoplasia* OR “malignant neoplasm*” OR malignanc*)) AND TS=(“Chinese medicine” OR “traditional Chinese medicine” OR TCM OR “herbal medicine” OR “botanical medicine” OR “material medicine” OR decoction OR tang OR powder OR san OR pill OR extract OR “Chinese proprietary medicine” OR “Chinese patent medicine” OR granules OR plaster OR plasters OR “oral liquid” OR “oral liquids” OR gels OR capsule OR syrup))).

Initially, 12558 articles were retrieved from the WOSCC database using keywords related to “traditional Chinese medicine,” “immunotherapy,” and “cancer,” with a time range from January 15, 2015, to January 15, 2025, and restricted to English-language literature. During the screening process, 3,483 articles were excluded for reasons such as non-compliance with literature type (e.g., conference abstracts, case reports), non-English language, and publication dates outside the specified range. A further 6616 articles were removed due to weak relevance to cancer treatment, Chinese medicine, or immunotherapy. Following these exclusions, 2459 valid articles remained for analysis. Data visualization and analysis were conducted using Origin 2018, CiteSpace(version 6.1.4), VOSviewer(version 1.6.17), and R software(version 3.6.3), focusing on institutions, countries, authors, journals, and keywords. A literature screening flowchart is provided in **Annex 1**.

### 2.2 Data analysis

To analyze the number of publications per year, Origin 2018 software was used in this study. Data visualization and the creation of scientific knowledge maps were performed using R software (version 3.6.3) with the bibliometrix package (version 4.0), VOSviewer (version 1.6.17), and CiteSpace (version 6.1.4). To ensure the accuracy and reliability of the data, data extraction and analysis were conducted independently by two authors.

VOSviewer was employed to visualize country/institution collaboration networks, source co-citation analysis, and keyword co-occurrence analysis. For the collaboration network analysis, the following parameters were set: a minimum of five publications for countries and a minimum of five publications for institutions. In the source co-citation analysis, the minimum number of citations for a source was set to 150. For the keyword co-occurrence analysis, the parameters included a minimum of 20 keyword occurrences. The following terms were excluded from the analysis: “cancer,” “cells,” “traditional Chinese medicine,” “immunomodulatory,” “mechanism,” “responses,” “immune response,” “immune,” “medicine,” “cell,” and “immune function.” These terms were already included in the search query, and their exclusion helps to better understand current research trends by avoiding redundancy and repetitive analysis.

The Impact Factor (IF) data for journals was obtained from the 2023 Journal Citation Reports (JCR) and used to assess the academic influence of the journals.

## 3 Results

### 3.1 Literature overview of TCM treats cancer by regulating the immune system

**Figure 1:**
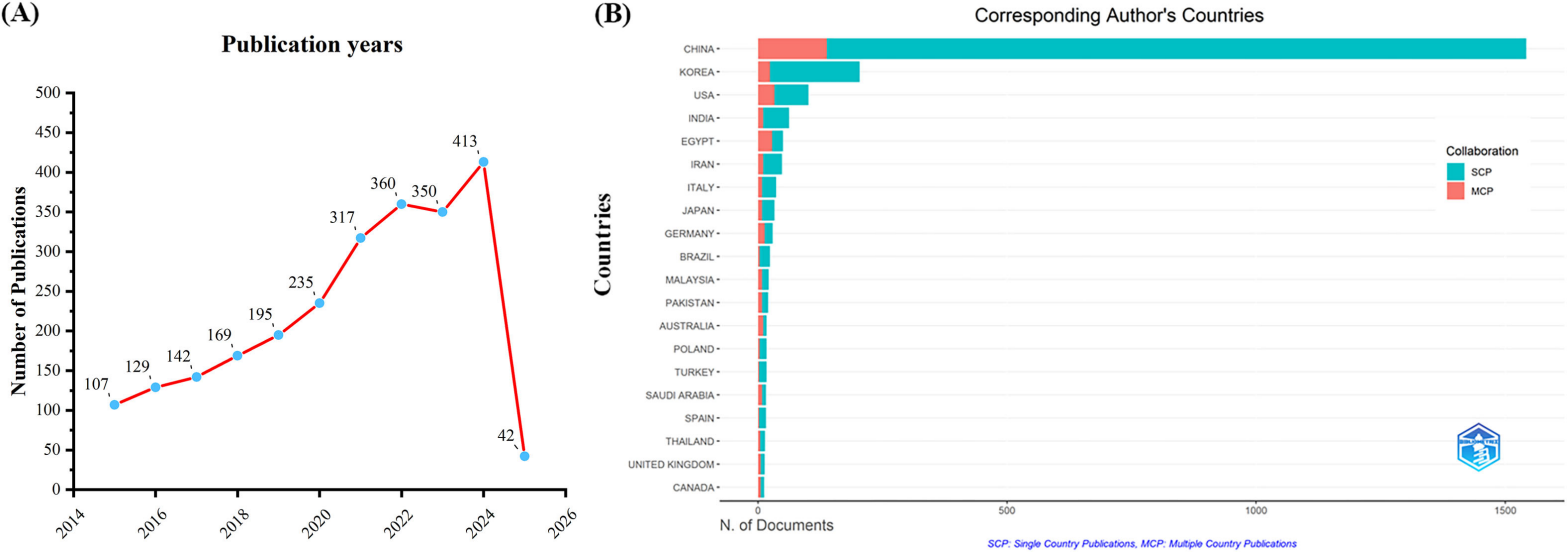
Trends in annual publication outputs TCM in treating cancer by regulating the immune system. **(A)** Trends of annual publication outputs. **(B)** Distribution of corresponding authors’ countries and cooperation.

A total of 12558 articles were initially collected from the WoSCC database. After removing duplicates and retracted articles, 2459 valid articles remained. As shown in **Figure 1A**, the number of publications related to TCM for cancer treatment through immune system modulation has steadily increased from 2015 to 2024, indicating a growing interest in this field over the years. A notable observation is the sharp increase in the number of publications after 2020, which may be associated with the surge in healthcare demand driven by the COVID-19 pandemic. Overall, this upward trend can be attributed to the growing global interest in tumor immunotherapy, advances in immunology and omics technologies, and increasing evidence supporting the immunomodulatory effects of TCM. In addition, national policy support, clinical demand for low-toxicity adjuvant therapies, and the internationalization of TCM research have further contributed to the academic output in this field.

Analysis of publication volume by the corresponding author’s country of origin reveals that China (n = 1,542) leads in publication output, followed by South Korea (n = 204), the United States (n = 101), India (n = 62), and Egypt (n = 50). Among the top five countries in terms of publication volume, China and South Korea exhibit relatively low cross-border publication proportions (MCPs), at 8.9% and 11.8%, respectively. These MCPs are significantly lower than those of the United States and Egypt, where the MCP ratios are 32.7% and 56.0%, respectively (**Figure 1B**, **Table 1**). This indicates that research in China and South Korea is predominantly focused on domestic publications, while the United States and Egypt demonstrate a stronger inclination toward international collaboration and transnational academic exchange.

**Table 1:** Most relevant countries by corresponding authors of Application of TCM in treating cancer by regulating the immune system.

Country	Articles	SCP	MCP	Freq	MCP_Ratio
China	1542	1404	138	0.627	0.089
Korea	204	180	24	0.083	0.118
USA	101	68	33	0.041	0.327
India	62	52	10	0.025	0.161
Egypt	50	22	28	0.02	0.56
Iran	48	38	10	0.02	0.208
Italy	36	28	8	0.015	0.222
Japan	33	25	8	0.013	0.242
Germany	29	16	13	0.012	0.448
Brazil	24	21	3	0.01	0.125
Malaysia	21	13	8	0.009	0.381
Pakistan	20	12	8	0.008	0.4
Australia	17	7	10	0.007	0.588
Poland	17	14	3	0.007	0.176
Turkey	17	15	2	0.007	0.118
Saudi Arabia	16	8	8	0.007	0.5
Spain	16	14	2	0.007	0.125
Thailand	14	10	4	0.006	0.286
United kingdom	13	8	5	0.005	0.385
Canada	12	7	5	0.005	0.417
Colombia	10	7	3	0.004	0.3
Mexico	10	9	1	0.004	0.1
Indonesia	8	4	4	0.003	0.5
Russia	7	6	1	0.003	0.143

SCP, Single Country Publications; MCP, Multiple Country Publications; MCP_Ratio=MCP/Articles.

**Figure 2:**
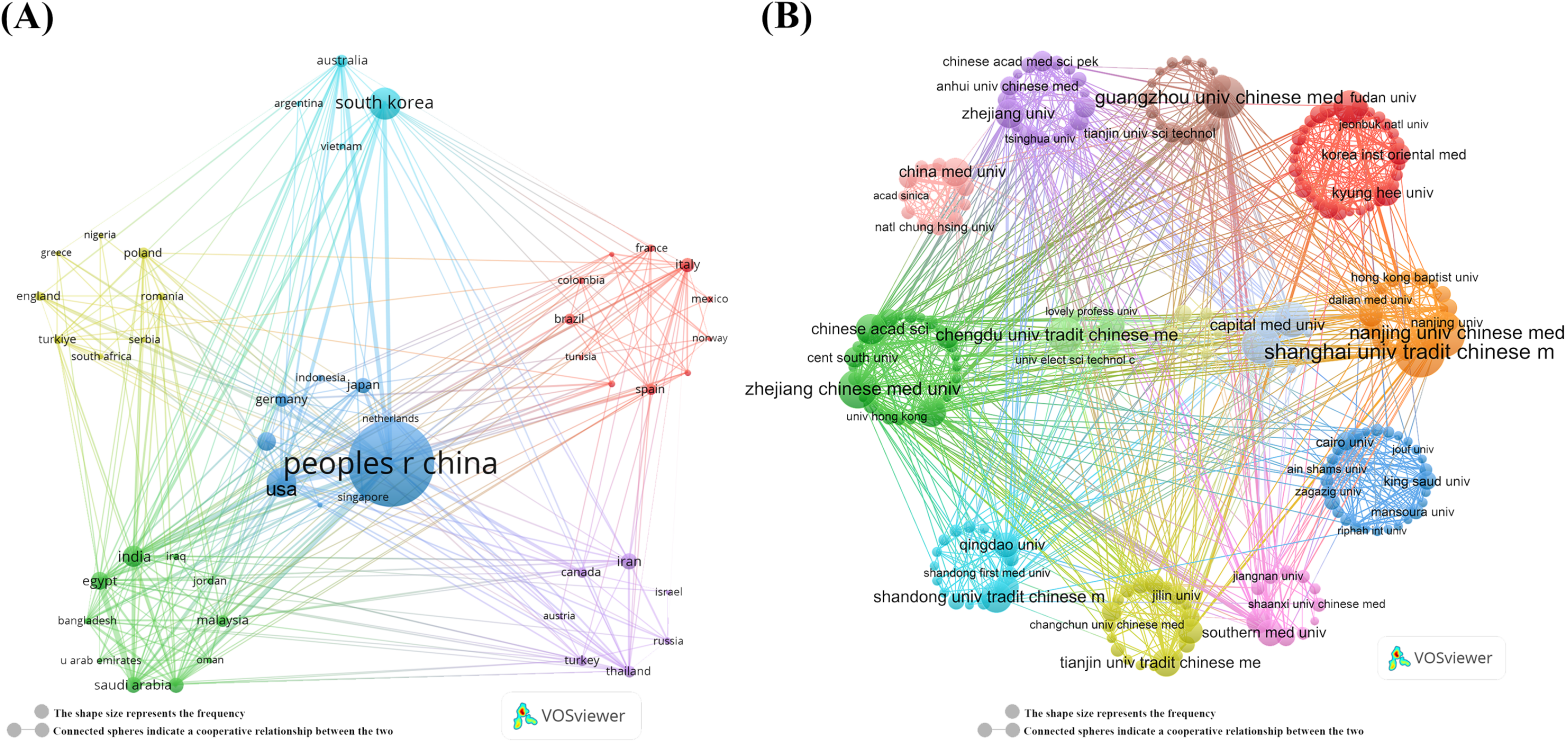
Map of countries/regions and institutions TCM in treating cancer by regulating the immune system. **(A)** Map of cooperation between different countries. **(B)** Map of cooperation between different institutions.

Further analysis of the collaboration network, depicted in **Figure 2A**, reveals that China (links:34) has extensive research collaborations with other countries, particularly in Asia, Europe, and North America. The visualized collaboration network highlights the Shanghai University of Traditional Chinese Medicine (n = 86) and Nanjing University of Chinese Medicin (n = 72) as key players in global research collaboration, establishing them as important centers of research collaboration (**Figure 2B**, **Table 2**). This underscores the critical role of TCM treats cancer by regulating the immune system, particularly in fostering international collaboration.

**Table 2:** Most relevant affiliations of Application of TCM in treating cancer by regulating the immune system.

Affiliation	Articles
Shanghai Univ Tradit Chinese Med	86
Nanjing Univ Chinese Med	72
Guangzhou Univ Chinese Med	65
Zhejiang Chinese Med Univ	56
Beijing Univ Chinese Med	55
Chengdu Univ Tradit Chinese Med	49
China Acad Chinese Med Sci	47
Fudan Univ	44
Shandong Univ Tradit Chinese Med	39
Chinese Acad Sci	37
China Med Univ	34
Sun Yat Sen Univ	34
Zhejiang Univ	34
Capital Med Univ	33
Tianjin Univ Tradit Chinese Med	32
Southern Med Univ	30
Kyung Hee Univ	27
Shanghai Jiao Tong Univ	27
Sichuan Univ	27
Qingdao Univ	26
Shanghai Univ Tradit Chinese Med	86
Nanjing Univ Chinese Med	72
Guangzhou Univ Chinese Med	65
Zhejiang Chinese Med Univ	56
Beijing Univ Chinese Med	55

### 3.2 Analysis of journals and co-citation journals

This study employed R software (version 3.6.3) with the Bibliometrix and ggplot2 packages to analyze journals in the field of TCM treats cancer by regulating the immune system. The analysis focused on identifying journals with the highest number of published articles and the most citations. Additionally, VOSviewer (version 1.6.17) was used for co-citation analysis. The results showed that 2,459 documents were distributed across 606 academic journals.

**Figure 3:**
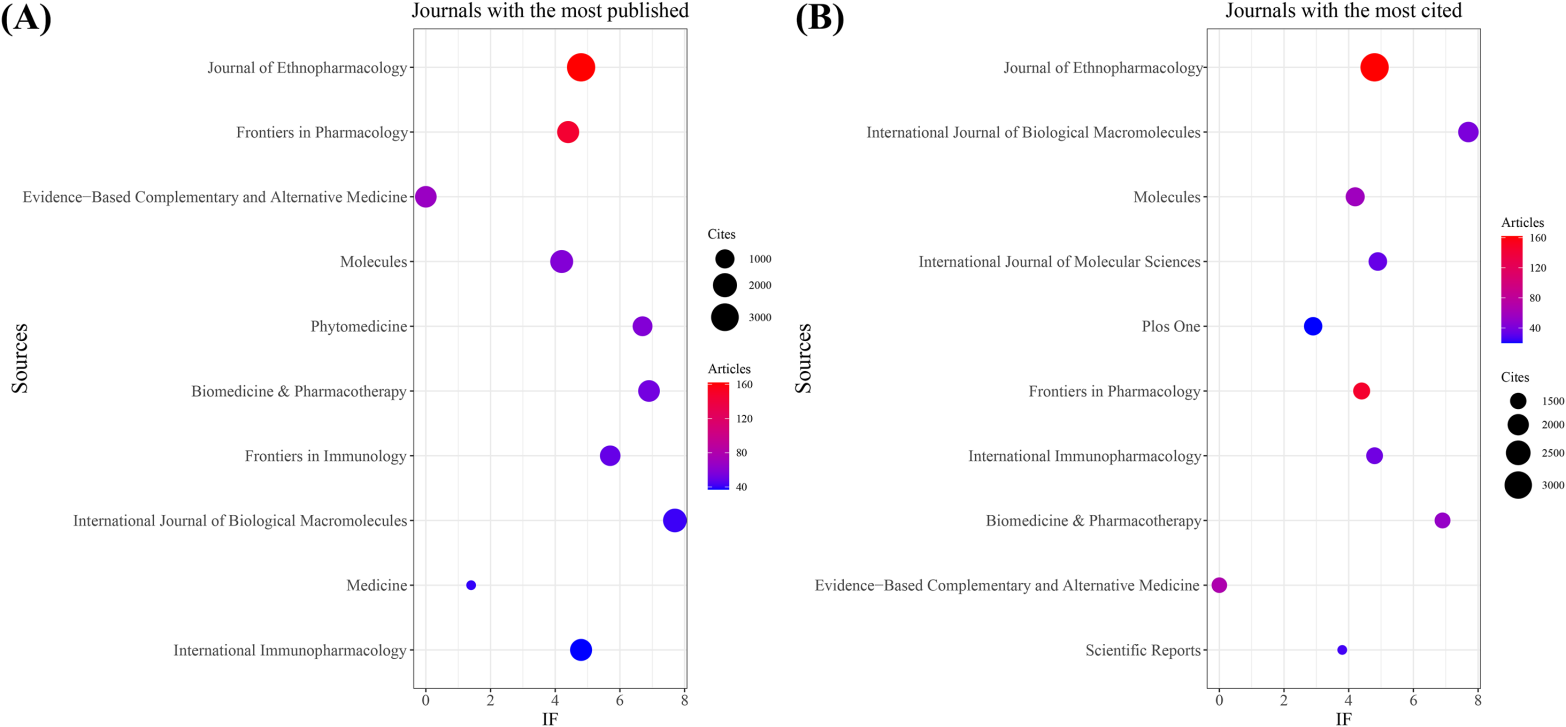
Journal with the largest number of articles published and the journal with the largest number of citations. **(A)** Journal with the largest number of articles published. **(B)** Journals with the largest number of citations.

As shown in **Table 3** and **Figure 3A**, the journal with the largest number of published articles is *Journal of Ethnopharmacology* (n = 162, IF = 4.8), followed by *Frontiers in Pharmacology* (n = 146, IF = 4.4), *Evidence-Based Complementary and Alternative Medicine* (n = 68, IF = 0), *Molecules* (n = 58, IF = 4.2), and *Phytomedicine* (n = 58, IF = 6.7). These journals have significant impact in the fields of TCM treats cancer by regulating the immune system, reflecting key research trends in the field.

**Table 3:** Top 10 journals with the most published articles.

Journal	Documents	IF (2023)	Cites
Journal of Ethnopharmacology	162	4.8	3177
Frontiers in Pharmacology	146	4.4	1545
Evidence-based Complementary and Alternative Medicine	68	0	1442
Molecules	58	4.2	1738
Phytomedicine	58	6.7	1122
Biomedicine & Pharmacotherapy	53	6.9	1465
Frontiers in Immunology	49	5.7	1245
International Journal of Biological Macromolecules	41	7.7	1901
Medicine	40	1.4	252
International Immunopharmacology	37	4.8	1534

IF, Impact Factor.

In terms of citation count, **Table 4** and **Figure 3B** show that *Journal of Ethnopharmacology* ranks first (n = 3,177, IF = 4.8), followed by *International Journal of Biological Macromolecules* (n = 1,901, IF = 7.7), *Molecules* (n = 1,738, IF = 4.2), *International Journal of Molecular Sciences* (n = 1,686, IF = 4.9), and *PLOS ONE* (n = 1,679, IF = 2.9). The citation frequency of these journals highlights their academic influence and the broad dissemination of their research.

**Table 4:** Top 10 journals with the most cited journals.

Journal	Cites	IF (2023)	Document
Journal of Ethnopharmacology	3177	4.8	162
International Journal of Biological Macromolecules	1901	7.7	41
Molecules	1738	4.2	58
International Journal of Molecular Sciences	1686	4.9	34
Plos One	1679	2.9	20
Frontiers in Pharmacology	1545	4.4	146
International Immunopharmacology	1534	4.8	37
Biomedicine & Pharmacotherapy	1465	6.9	53
Evidence-based Complementary and Alternative Medicine	1442	0	68
Scientific Reports	1252	3.8	26

IF, Impact Factor.

**Figure 4:**
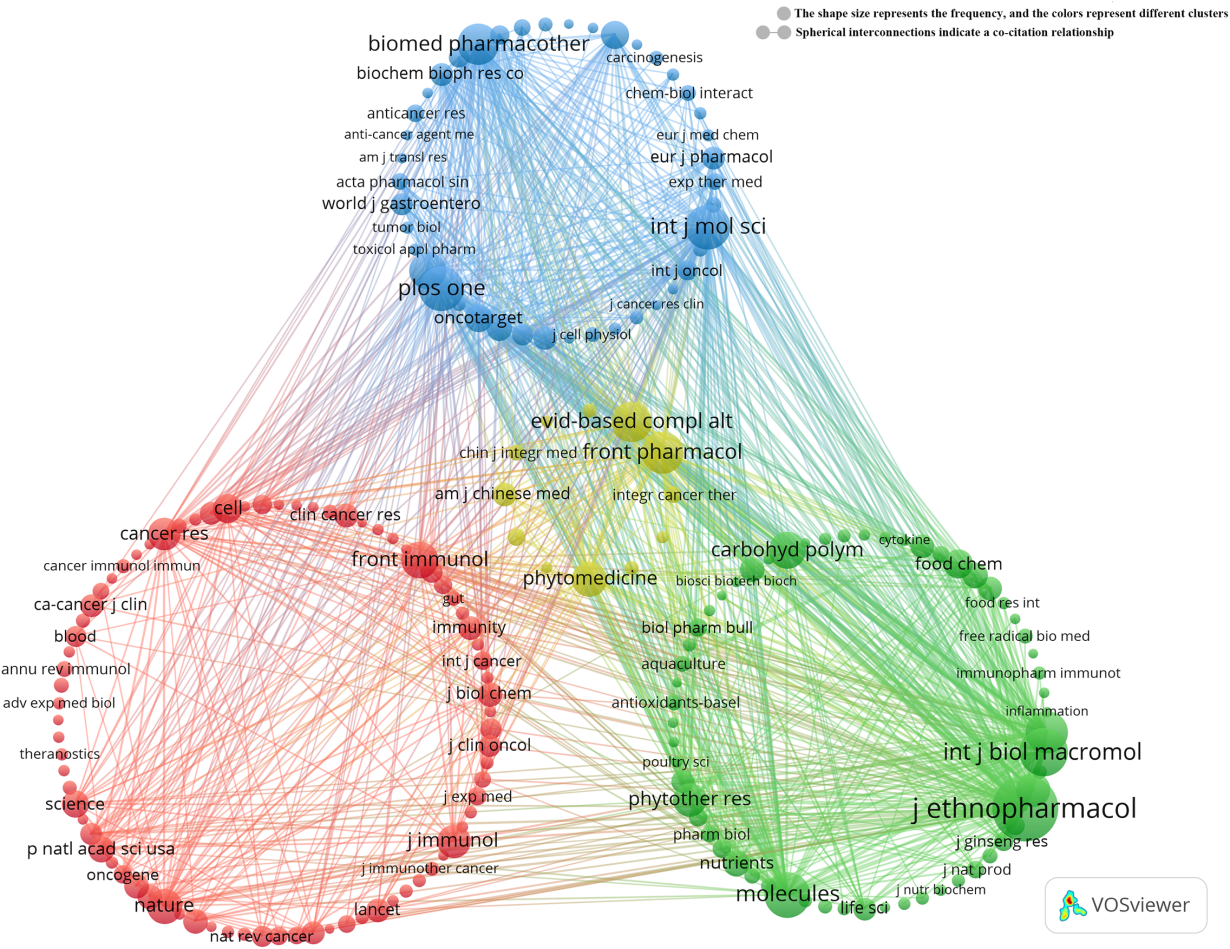
Co-cited journals involved in TCM in treating cancer by regulating the immune system.

Co-citation analysis further reveals that *Journal of Ethnopharmacology* and *Frontiers in Pharmacology* serve as central hubs for research on TCM treats cancer by regulating the immune system (**Figure 4**). These journals, along with *Molecules*, lead in both publication volume and citation count. These findings suggest that *Journal of Ethnopharmacology*, *Frontiers in Pharmacology*, and *Molecules* are among the most representative and influential journals in this field.

However, despite the strong performance of these journals in terms of publications and citations, it is noteworthy that the concentration of research related to TCM treats cancer by regulating the immune system remains relatively low in these leading journals. This phenomenon highlights a gap in the research depth and quality in the field, indicating the need for a stronger focus on fostering deeper research, interdisciplinary collaboration, and innovative studies, particularly in the area of TCM for cancer treatment.

### 3.3 Citation burst

**Figure 5:**
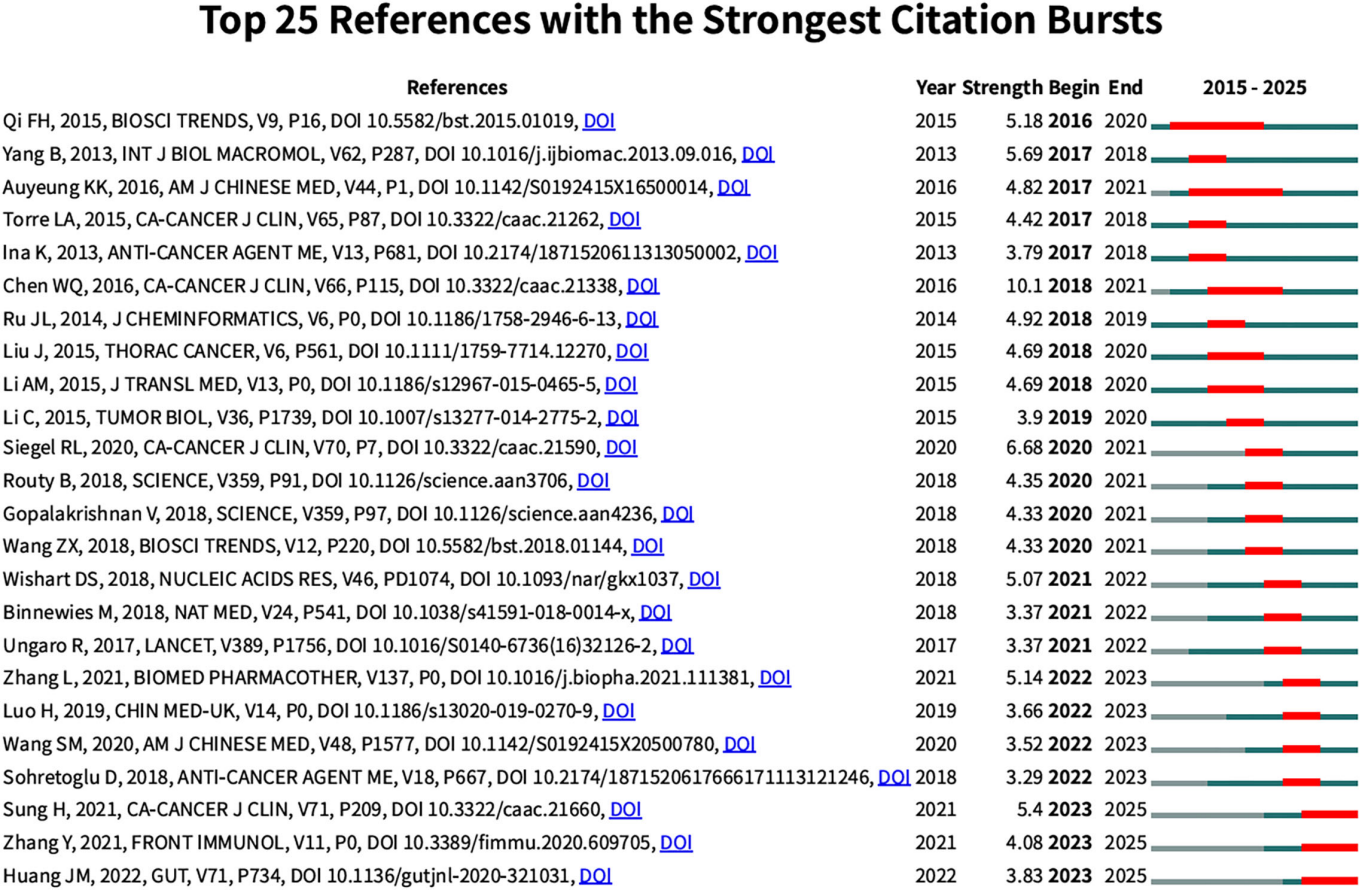
Top 25 references with the strongest citation bursts on TCM in treating cancer by regulating the immune system.

To identify citation bursts, we used CiteSpace to analyze the top 25 papers with citation bursts lasting at least two years. A total of 36 papers with significant citation bursts were identified, with the top 25 shown in **Figure 5**. The titles of these citations and their respective DOIs are listed in **Annexes 2**. The three papers with the most active citation bursts are:(1)”Cancer Statistics in China, 2015” (intensity: 10.1)(2)”Cancer Statistics, 2020” (intensity: 6.68)(3)” Astragalus membranaceus: A Review of its Protection Against Inflammation and Gastrointestinal Cancers” (intensity: 5.69).

Further analysis reveals that the following three articles have experienced a notable surge in citations in recent years:(1)”Ginseng Polysaccharides Alter the Gut Microbiota and Kynurenine/Tryptophan Ratio, Potentiating the Antitumor Effect of Anti-PD-1/PD-L1 Immunotherapy” (intensity: 3.83)(2)”Research Status and Molecular Mechanism of the TCM and Antitumor Therapy Combined Strategy Based on Tumor Microenvironment” (intensity: 4.08)(3)”Global Cancer Statistics 2020: GLOBOCAN Estimates of Incidence and Mortality Worldwide for 36 Cancers in 185 Countries” (intensity: 5.4).

The surge in citations of these articles underscores their importance in tumor immunotherapy, the combination of TCM with antitumor therapy, and the tumor microenvironment.

Through citation burst analysis, several key research frontiers and hotspots in the field of TCM treats cancer by regulating the immune system were identified, particularly regarding its role in cancer treatment. These research directions include: (1) Clinical Research and Application of TCM in Cancer Treatment, which focuses on the use of TCM in clinical practice, especially during the perioperative period; (2) Immune and Inflammatory Response Mechanisms of TCM in Cancer Treatment, which explores how TCM can enhance cancer immunotherapy by modulating immune responses and inflammatory pathways; and (3) The Influence of Traditional Chinese Medicine on Immune Function, examining how TCM affects overall immune function in cancer therapy.

### 3.4 Keyword clustering and topic evolution

**Figure 6:**
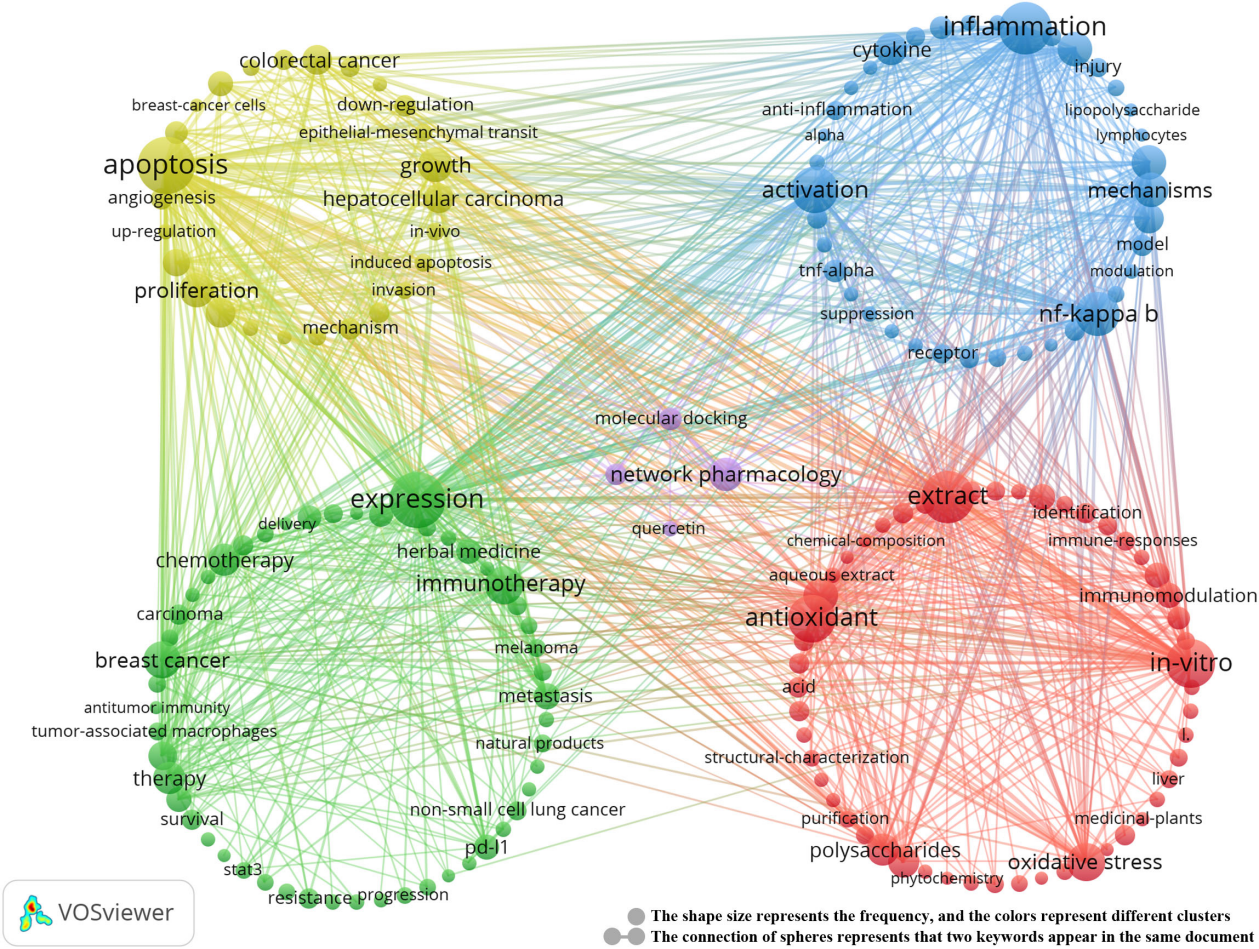
Keyword co-occurrence map of publications on TCM in treating cancer by regulating the immune system.

Keyword clustering analysis is crucial for identifying core topics and emerging trends in a research field. In this study, we used the VOSviewer tool to analyze 10,299 keywords and selected the top 20 keywords that appeared more than 20 times (**Table 5**). These keywords represent the primary research directions in the field of TCM treats cancer by regulating the immune system The most frequent keyword was “apoptosis” (n = 352), followed by “expression” (n = 343), “inflammation” (n = 298), “extract” (n = 294), “*in-vitro*” (n = 255), “activation” (n = 235), “antioxidant” (n = 248), and “NF-kappa B” (n = 216).Through cluster analysis, we identified five major research clusters, shown in **Figure 6**:

**Table 5:** Top 20 keywords related to TCM in treating cancer by regulating the immune system.

Rank	Keywords	Occurrences
1	apoptosis	352
2	expression	343
3	inflammation	298
4	extract	294
5	*in-vitro*	255
6	activation	235
7	antioxidant	248
8	nf-kappa b	216
9	breast cancer	146
10	immunotherapy	163
11	proliferation	130
12	growth	136
13	oxidative stress	156
14	inhibition	134
15	antitumor	133
16	macrophage	133
17	mechanisms	135
18	therapy	116
19	pathway	99
20	polysaccharide	106

Biological Activity and Immunomodulation of TCM (red dots): (1) This cluster includes 52 keywords, such as anti-inflammatory, antioxidant, anti-tumor, flavonoids, polysaccharides, and saponins, highlighting the potential of TCM in immunomodulation and anti-tumor effects. (2) Cancer Immunotherapy and Clinical Application (yellow dots): Comprising 51 keywords, this cluster focuses on cancer immunotherapy, including specific cancers like breast and lung cancer, chemotherapy, and immune checkpoint inhibitors, reflecting the latest advances in cancer immunotherapy. (3) TCM Regulates Immune Inflammation and Immune Diseases (green dots): Containing 35 keywords, this cluster explores pathways such as cytokines, immune cells, inflammation, NF-kappa B, and TNF-alpha, emphasizing TCM’s unique role in treating immune diseases. (4) Mechanisms of Action of TCM in Cancer Cells (blue dots): With 26 keywords, this cluster includes terms like angiogenesis, apoptosis, tumor cells, cancer cells, natural products, and cancer treatment mechanisms, illustrating the complexity of TCM’s mechanisms in cancer treatment. (5) Molecular Docking and Network Pharmacology (purple dots): This cluster includes 4 keywords focusing on molecular docking, network pharmacology, and compounds such as quercetin, reflecting the growing interest in applying these techniques in TCM cancer immunotherapy. For detailed information on the keywords within each cluster, refer to **Annexes 3**.

**Figure 7:**
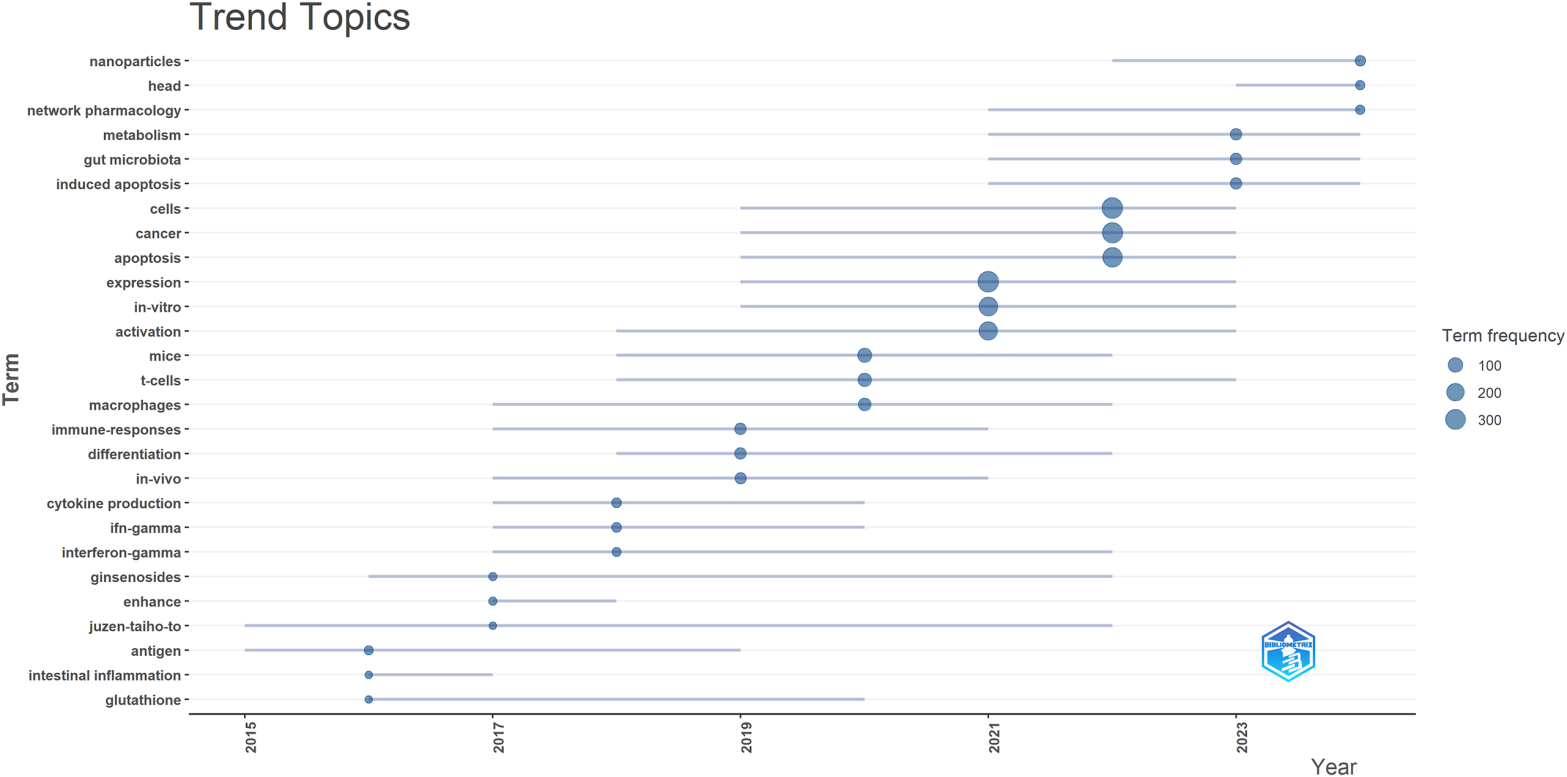
Trend topics on TCM in treating cancer by regulating the immune system.

To predict future trends in this field, the Bibliometrix package in R software was used to create a topic evolution diagram (**Figure 7**), enabling a chronological examination of specific research topics and revealing the evolutionary trajectory of TCM in cancer treatment by regulating the immune system. The trend topic map in **Figure 7** highlights key research areas, such as nanoparticles, head, and network pharmacology. The analysis identifies three main research directions in TCM treats cancer by regulating the immune system: (1) Mechanism of Action of TCM treats cancer by regulating the immune system, with ongoing research focusing on understanding how TCM influences cancer immunotherapy at a fundamental level; (2) Combination of Cancer Immunotherapy with TCM, as there is growing interest in combining TCM with conventional cancer immunotherapy in clinical practice; and (3) Application of Molecular Docking and Network Pharmacology in TCM Cancer Immunotherapy, where the use of molecular docking and network pharmacology has become a key frontier in studying TCM’s effects on cancer treatment.

## 4 Discussion

### 4.1 Overview

This study analyzed a total of 2,459 articles published between 2015 and 2025. The data revealed an overall upward trend in publications related to how TMC treats cancer by regulating the immune system.

China remains the largest contributor to research on TCM treats cancer by regulating the immune system, reflecting the country’s deep-rooted history in traditional medicine. The prominence of Chinese researchers underscores the ongoing significance of TCM in China. China and its institutions have played a leading role in this field primarily due to the historical, cultural, and policy-driven foundations of traditional Chinese medicine (TCM). TCM has been an integral part of China’s healthcare system for thousands of years, with well-established theories and practices that emphasize holistic and immune-based approaches to disease management. In recent years, the Chinese government has significantly increased investment in TCM modernization and integration with Western medicine, particularly through national strategies such as the “Healthy China 2030” initiative and inclusion of TCM in national cancer treatment guidelines. Substantial research funding, dedicated TCM research institutes, and supportive regulatory frameworks have empowered Chinese institutions to lead large-scale, interdisciplinary studies exploring the immunomodulatory role of TCM in cancer therapy. Consequently, China has emerged as a global hub for research in this domain.

Of the 2459 articles published across 606 journals, high-impact journals such as *Journal of Ethnopharmacology*, *Frontiers in Pharmacology*, and *Molecules* have played a pivotal role in advancing the field. The *Journal of Ethnopharmacology*, in particular, stood out due to its high volume of published articles and citations, reinforcing its central role in the dissemination of TCM-related research. This journal’s significant influence highlights its importance in academic discussions, particularly regarding TCM’s role in cancer immunotherapy and treatment.

### 4.2 Hotspots and development trends

Through a comprehensive analysis of literature clustering, keyword frequency, keyword clustering, and theme evolution, the main research hotspots and development trends of TCM treats cancer by regulating the immune system have been identified. The key research areas include the following three points:

#### 4.2.1 TCM treats cancer by regulating the immune system in regulating immunotherapy

The mechanisms through which TCM regulates cancer immunotherapy are multifaceted, involving multiple targets and components to enhance anti-tumor immune responses. Below are the detailed mechanisms, along with specific examples:

(1) Regulation of Immune Cell Function

TCM enhances anti-tumor immunity by regulating the function of various immune cells, including T-cells, NK cells, and macrophages. For instance, *Astragalus* polysaccharides stimulate CD4+ and CD8+ T-cells proliferation and cytokine secretion via the JAK/STAT pathway, thereby enhancing T-cells-mediated anti-tumor responses (16, 17). Similarly, ginsenosides in *Ginseng* promote T-cells proliferation and improve tumor-specific activity, thereby enhancing the effectiveness of cancer immunotherapy (18). Additionally, TCM strengthens the activity of NK cells, which are crucial for directly killing tumor cells. *Ganoderma lucidum* polysaccharides activate NK cells through Toll-like receptor (TLR) signaling, stimulating the release of cytokines such as IFN-γ and TNF-α, which are especially beneficial in liver and lung cancer treatments (19, 20). *Danshen* (Salvia miltiorrhiza) also enhances NK cell cytotoxicity, particularly in breast cancer (21). Macrophages, key players in immune defense, can be activated by TCM to further enhance their anti-tumor activity (22). *Lycium barbarum* (Goji berries) boosts macrophage phagocytosis and cytokine secretion, while *Astragalus* stimulates macrophage activity by promoting pro-inflammatory cytokines like TNF-α and IL-6, improving immune responses within the tumor microenvironment, particularly in liver and lung cancer (23, 24)

(2) Inhibition of Tumor Immune Escape Mechanisms

TCM plays a crucial role in inhibiting tumor immune escape mechanisms, which often reduce the effectiveness of immunotherapy (25). Tumor cells evade immune surveillance through various immune escape strategies, and TCM helps regulate these mechanisms through multiple pathways. One key approach is the regulation of regulatory T-cells (Tregs), which suppress anti-tumor immune responses by secreting immunosuppressive cytokines such as TGF-β and IL-10 (26). TCM ingredients such as curcumin and resveratrol can alleviate the immunosuppressive effects of Tregs, thereby enhancing the immune system’s ability to fight tumors (27). For instance, curcumin, when combined with chemotherapy, has been shown to reduce Tregs-mediated immunosuppression, improving the overall efficacy of immunotherapy (28). Additionally, TCM can inhibit the production of immunosuppressive factors like TGF-β and IL-10, which are prominent in the tumor microenvironment. Herbs such as curcumin and resveratrol have been found to reduce TGF-β levels, enhancing T-cells anti-tumor activity, reducing immune escape, and further improving the efficacy of immunotherapy (29, 30). Furthermore, TCM can modulate tumor-associated macrophages (TAMs), which are key regulators of tumor immune escape. TAMs predominantly exhibit an M2 phenotype within the tumor microenvironment, characterized by the secretion of anti-inflammatory cytokines (e.g., IL-10, TGF-β), expression of immune checkpoint molecules, and promotion of regulatory T cell (Treg) recruitment. These features collectively contribute to an immunosuppressive milieu that facilitates tumor immune evasion, growth, and metastasis (31). TCM has been shown to reprogram TAMs toward the M1 phenotype, which is associated with pro-inflammatory cytokine production (e.g., IL-12, TNF-α), enhanced antigen presentation, and activation of cytotoxic T lymphocytes. This phenotypic shift disrupts the immunosuppressive microenvironment, restores anti-tumor immunity, and impairs the mechanisms by which tumor cells escape immune surveillance. For example, herbs such as *Lycium barbarum* and *Astragalus membranaceus* have been reported to promote M1 polarization of TAMs, thereby inhibiting tumor progression and enhancing immune-mediated tumor clearance (32, 33).

(3) Regulation of Immune-Related Signaling Pathways

TCM enhances immune responses and inhibits tumor growth by modulating immune-related signaling pathways, which are pivotal in tumor immune escape and immune tolerance (34, 35). TCM holds significant potential in regulating these pathways, thereby improving the efficacy of immunotherapy. For example, TCM can modulate the NF-κB pathway, which plays a critical role in both immune escape and tumor cell growth. Herbs such as *Astragalus* and *Salvia* miltiorrhiza have been shown to inhibit NF-κB activation, reduce tumor cell tolerance to immune responses, and enhance anti-tumor immunity (36, 37). *Astragalus* polysaccharide, an active ingredient of Astragalus, has been demonstrated to significantly inhibit NF-κB activation, enhance immune cell anti-tumor functions, reduce immune escape, and promote tumor control (38). Furthermore, TCM can regulate the JAK/STAT signaling pathway, which plays a central role in immune cell function and tumor immune escape (39). Studies have shown that compounds like curcumin and resveratrol can inhibit JAK/STAT pathway activation, reduce tumor cell evasion of immune surveillance, and enhance immune cell-mediated anti-tumor responses (40, 41). Curcumin, in particular, regulates the JAK/STAT3 pathway, inhibits tumor growth, and improves immunotherapy efficacy (42). Additionally, TCM can influence the MAPK signaling pathway, which is linked to tumor cell proliferation, survival, and immune response (43). Herbs like *Lycium barbarum* (*wolfberry*) and *Ganoderma lucidum* have been shown to regulate the MAPK pathway, enhancing the anti-tumor function of immune cells and inhibiting tumor cell proliferation (44, 45). For instance, *Lycium barbarum* significantly inhibits tumor growth and enhances immune cell activity by modulating the activity of ERK1/2 proteins in the MAPK pathway, thereby improving the overall effectiveness of immunotherapy (46).

In summary, TCM offers a multifaceted approach to cancer immunotherapy, enhancing immune cell activity, inhibiting immune escape mechanisms, and modulating key signaling pathways, thereby improving the overall effectiveness of immunotherapy.

#### 4.2.2 Clinical application of cancer immunotherapy combined with traditional Chinese medicine

Cancer immunotherapy, particularly the use of immune checkpoint inhibitors (e.g., PD-1/PD-L1 antibodies), has significantly advanced cancer treatment by activating immune cells and enhancing their ability to target and eliminate tumor cells (47). Despite its success, challenges such as treatment resistance, immune-related side effects, and variations in treatment responses persist (48). Thus, improving the efficacy of immunotherapy, reducing adverse effects, and prolonging the duration of therapeutic responses have become critical objectives in clinical cancer treatment.

TCM, with its multi-component and multi-target approach, can complement cancer immunotherapy to address these challenges. TCM can enhance the effectiveness of immunotherapy, mitigate its side effects, and improve patients’ quality of life by regulating various immune system components (49). The synergistic effects of combining TCM with immunotherapy can lead to improved therapeutic outcomes. The following section delves into the specific ways in which TCM can enhance the efficacy of cancer immunotherapy.

(1) Improving the efficacy of immunotherapy

The success of immunotherapy relies on activating the immune system to accurately identify and eliminate tumor cells, and TCM plays a crucial role in supporting this process through multiple mechanisms. Numerous studies have shown that TCM significantly enhances the effectiveness of immunotherapy by improving T-cells function. Herbs such as *Astragalus, Ganoderma lucidum*, and *Wolfberry* promote immune responses by regulating T-cells activity and cytokine production (23, 50, 51). For instance, *Astragalus* polysaccharide, an active ingredient in *Astragalus*, activates T-cells and enhances their anti-tumor function by stimulating the secretion of immune factors such as IL-2 and IFN-γ (50). Clinical studies have demonstrated that combining *Astragalus* with anti-PD-1 antibodies enhances immune cell activity and strengthens anti-tumor immune responses (52). This combination therapy has shown positive results in treating liver cancer and non-small cell lung cancer (53). Natural Killer (NK) cells are essential for immune defense, as they directly recognize and kill tumor cells. TCMs, including *Ganoderma lucidum* and *Salvia miltiorrhiza*, enhance NK cell activity and, consequently, their anti-tumor effects (54, 55). The polysaccharide components in *Ganoderma lucidum* significantly boost NK cell activity and tumor-killing ability. When used in conjunction with immune checkpoint inhibitors, *Ganoderma lucidum* extracts can significantly increase NK cell activity and improve immune responses. For example, combining Ganoderma lucidum with anti-PD-1 antibodies significantly improved the efficacy of immunotherapy in lung cancer patients (56). Moreover, TCM supports the persistence and enhancement of immune responses by modulating the secretion of key immune factors. Herbs such as *Wolfberry* and *Astragalus* further stimulate immune cell activation by promoting the production of cytokines such as IL-6 and IL-2 (57, 58). These immunomodulatory properties enhance the overall effectiveness of immunotherapy by stimulating various components of the immune system. In conclusion, TCM improves immunotherapy outcomes by enhancing immune cell function, bolstering immune responses, and prolonging therapeutic effects, making it a valuable complementary approach in cancer treatment.

(2) Alleviating the Side Effects of Immunotherapy

Although cancer immunotherapy has made significant advances in therapeutic efficacy, its side effects remain a major challenge in clinical practice (5). Immune-related adverse reactions, such as immune pneumonia, hepatitis, colitis, and rashes, can occur during treatment, and some patients may even experience persistent inflammatory responses (59–62). TCM offers valuable support in alleviating these side effects through multiple mechanisms:

Immunotherapy can sometimes lead to the overactivation of immune cells, which in turn triggers immune-related adverse reactions such as immune pneumonia and hepatitis. Chinese herbs like resveratrol and astragalus possess potent anti-inflammatory and immunomodulatory properties that help mitigate these reactions. By regulating immune responses, these herbs prevent the immune system from attacking healthy tissues, thus alleviating side effects like rashes and liver toxicity. Resveratrol, a natural antioxidant, plays a critical role in inhibiting immune cell overactivation. It helps reduce immune-related liver damage and prevent immune hepatitis (63). Similarly, *Astragalus* supports immune regulation, reducing overactivation during immunotherapy. It protects normal tissues, enhances immune responses, and effectively controls inflammation and immune-related damage (64).

TCM also enhances the body’s tolerance to the side effects of immunotherapy by regulating the function of internal organs such as the spleen, stomach, and liver. By improving the functions of these organs, TCM optimizes the immune system and strengthens the body’s resilience during treatment. Herbs such as *Astragalus* and *Codonopsis* help improve the qi and blood functions of the spleen and stomach, boost overall immunity, and increase the body’s tolerance to the stress of immunotherapy (65). These herbs align with the TCM principle of strengthening the body’s “positive energy” to better combat external pathogens. Clinical studies have shown that *Astragalus*, when combined with chemotherapy or immunotherapy, can improve immune tolerance, reduce adverse reactions, and prolong the efficacy of immunotherapy (66).

Furthermore, TCM works by regulating internal organ function, balancing qi and blood, and enhancing immune function to restore overall health. This holistic approach not only boosts the effects of immunotherapy but also helps minimize its side effects. By methods such as replenishing qi and blood, regulating liver qi, and other therapeutic techniques, TCM helps patients regain strength and enhance immune function. It is effective in alleviating common immunotherapy side effects, such as fatigue, loss of appetite, and indigestion. These benefits are achieved through herbal treatments, acupuncture, and other holistic therapies, all of which improve patients’ quality of life and help them better tolerate treatment (67).

In summary, TCM plays a supportive role in cancer immunotherapy by managing and relieving side effects, improving overall health, and promoting better treatment outcomes. By combining TCM with modern immunotherapy, patients can better cope with treatment, improve their quality of life, and prolong their response time to treatment.

#### 4.2.3 Application of traditional Chinese medicine in treating various cancers through the immune system

In recent years, TCM has garnered increasing attention for its potential role in cancer immunotherapy. TCM has shown promising results in enhancing the body’s anti-tumor immune response and improving the tumor microenvironment, thus offering new avenues for cancer treatment. For instance, in colorectal cancer, herbs like *Astragalus*, *Codonopsis*, and *Ganoderma lucidum* have been found to bolster immune responses by modulating T-cells activity and boosting NK cell function (54, 68, 69). Clinical studies indicate that combining these herbs with chemotherapy can enhance immune cell activity and promote tumor cell apoptosis (70). Moreover, combining TCM with immune checkpoint inhibitors such as PD-1 antibodies has proven effective in amplifying immune responses and inhibiting tumor growth (71).

In addition, TCM is increasingly being incorporated into treatment strategies for cholangiocarcinoma, a challenging bile duct cancer (72). Herbs such as *Atractylodes macrocephala* and *Bupleurum chinense* enhance immune responses by stimulating T-cells activity and inhibiting immunosuppressive cells. These herbs improve the immune microenvironment, enhance NK cell activity, and reduce tumor growth (73, 74). In breast cancer, TCM helps regulate immune responses by promoting T-cells activation, NK cell proliferation, and macrophage function, and shows promise when combined with modern immunotherapies like PD-1 inhibitors. Clinical studies have demonstrated that TCM combined with immunotherapy can significantly enhance the immune system’s ability to fight breast cancer (75, 76).

For lung cancer, one of the most lethal cancers worldwide (77), herbs like *Ganoderma lucidum*, *bitter melon*, and *Codonopsis pilosula* improve NK and T-cells function, modulate the immune microenvironment, and enhance immune responses (78–80). Gastric cancer, another prevalent malignancy, benefits from TCM-enhanced immunotherapy. Herbs such as *Astragalus* and *Hedyotis diffusa* have shown positive effects by boosting T-cells and NK cell activity, inhibiting tumor metastasis, and modulating the immune environment (66, 81). Lastly, ovarian cancer treatment benefits from TCM’s immunomodulatory effects, with herbs like *Astragalus*, *Codonopsis pilosula*, and *Ganoderma lucidum* improving immune cell function and reducing tumor recurrence during chemotherapy (82, 83).

In conclusion, TCM’s ability to boost immune function, regulate the tumor microenvironment, and address challenges like immune escape and drug resistance positions it as a valuable complementary approach to modern cancer therapies, improving outcomes for patients across a range of cancers.

### 4.3 Limitations and future directions

#### 4.3.1 Limitations

(1) Data Source Limitations

This study primarily relies on the Web of Science (WoS) database, which may have overlooked relevant literature from other important databases such as PubMed, Scopus, and CNKI. While WoS plays a key role in bibliometric analysis, restricting the data source to only one database could limit the comprehensiveness of the research findings. However, the WoSCC provides comprehensive bibliometric indicators (e.g., h-index, citation counts) and structured metadata, which are essential for conducting rigorous bibliometric analyses. Its standardized format also facilitates cross-study comparisons. Limiting the data source to WoS ensures that our study can be replicated using a widely recognized and accessible database. Expanding to multiple databases may introduce variability due to indexing overlaps and inconsistencies. Including multiple databases such as Scopus and PubMed would significantly increase the complexity of data extraction and deduplication processes, potentially leading to errors. Several databases, such as PubMed, do not support the export of full records and cited references. Given the trade-off between data breadth and depth, we prioritize data depth. Moreover, in the context of medical research, there is a high degree of overlap between records indexed in WoS and those in databases like PubMed. Accordingly, we are confident that the results of our study are both methodologically sound and reliable.

(2) Language Bias

This study focused on analyzing English-language literature, which may have missed significant studies published in other languages (such as Chinese). This language bias could impact the completeness and accuracy of the research conclusions.

#### 4.3.2 Future research directions

Future research on TCM in cancer immunotherapy should focus on the following key areas to enhance its potential application:

(1) Mechanisms of Action of Active Ingredients

Further research should delve into the specific mechanisms by which active ingredients in TCM affect immune responses, inflammation, and the intestinal microbiota. Understanding these biological effects will help improve their clinical therapeutic applications and enhance their potential for cancer treatment.

(2) Efficacy and Safety in Diverse Populations

Future studies should assess the efficacy and safety of TCM across different patient populations with varying pathological characteristics and genetic backgrounds. This will ensure that TCM treatments are not only universally applicable but also personalized to better meet individual patient needs.

(3) Long-Term Effects of TCM

It is important to evaluate the long-term effects of TCM, including disease relief, improvements in patients’ quality of life, and any potential side effects. This will help determine the effectiveness of TCM in practical, long-term cancer care.

(4) Precision Cancer Treatment

Research should move toward precision cancer treatment, aiming to address the complex biological characteristics of cancer and improve treatment efficacy. The development of more targeted therapies using TCM could significantly enhance treatment outcomes.

(5) Combination Therapies with Modern Medicine

Exploring combinations of TCM with modern medicine, particularly Western medicine, could improve treatment efficacy. This approach offers a more comprehensive strategy for cancer treatment and may unlock new synergies in therapeutic regimens.

By addressing these key areas, future research will strengthen the scientific foundation of TCM, promote its integration into modern medicine, and advance its development as a viable treatment option in cancer therapy.

## 5 Conclusion

This study highlights the key research hotspots and emerging trends in TCM treats cancer by regulating the immune system. The main findings include:

(1) Global Attention to TCM Immunotherapy

There is growing global interest in the application of TCM in immunotherapy, with China, the United States, South Korea, Japan, and Brazil leading research efforts. International cooperation in this field is vibrant and driving significant progress.

(2) Important Journals

The *Journal of Ethnopharmacology* and *Frontiers in Pharmacology* are leading journals in TCM-based cancer immunotherapy research. The *Journal of Ethnopharmacology* is particularly influential, with the highest number of citations in this area.

(3) Mechanistic Insights

Regulation of immune cell function, inhibition of tumor immune escape mechanisms, and regulation of immune - related signaling pathways.

(4) Translational Focus

Recent research increasingly integrates molecular mechanism studies with clinical investigations, reflecting a growing emphasis on evidence-based application and translational relevance.

(5) Treatment Strategies

The combination of TCM with anti-inflammatory and antioxidant properties in cancer treatment has emerged as a key strategy to enhance the efficacy of immunotherapy. At the same time, the potential of TCM in alleviating the side effects of immunotherapy has garnered widespread attention.

This study broadens the research perspective in cancer immunotherapy, offering valuable insights into future research directions and clinical applications. Based on these findings, we recommend that future research focus on integrating TCM with advanced immunological and multi-omics technologies to clarify underlying mechanisms and enhance clinical translation. High-quality clinical trials and international, interdisciplinary collaboration should be prioritized to maximize scientific and therapeutic value. For policymakers, sustained investment, supportive regulatory frameworks, and initiatives that promote global cooperation are essential to facilitate the development and integration of TCM-based immunotherapies into standardized cancer treatment protocols.

## Data Availability

Publicly available datasets were analyzed in this study. This data can be found here: Web of Science.
