# Supplementary figures and images for: Bibliometric analysis of traditional Chinese medicine in cancer treatment via immune system modulation (2015–2025)
Source: Front Immunol. 2025 May 8;16:1581885. doi: 10.3389/fimmu.2025.1581885 (PMC12095241; doi:10.3389/fimmu.2025.1581885)

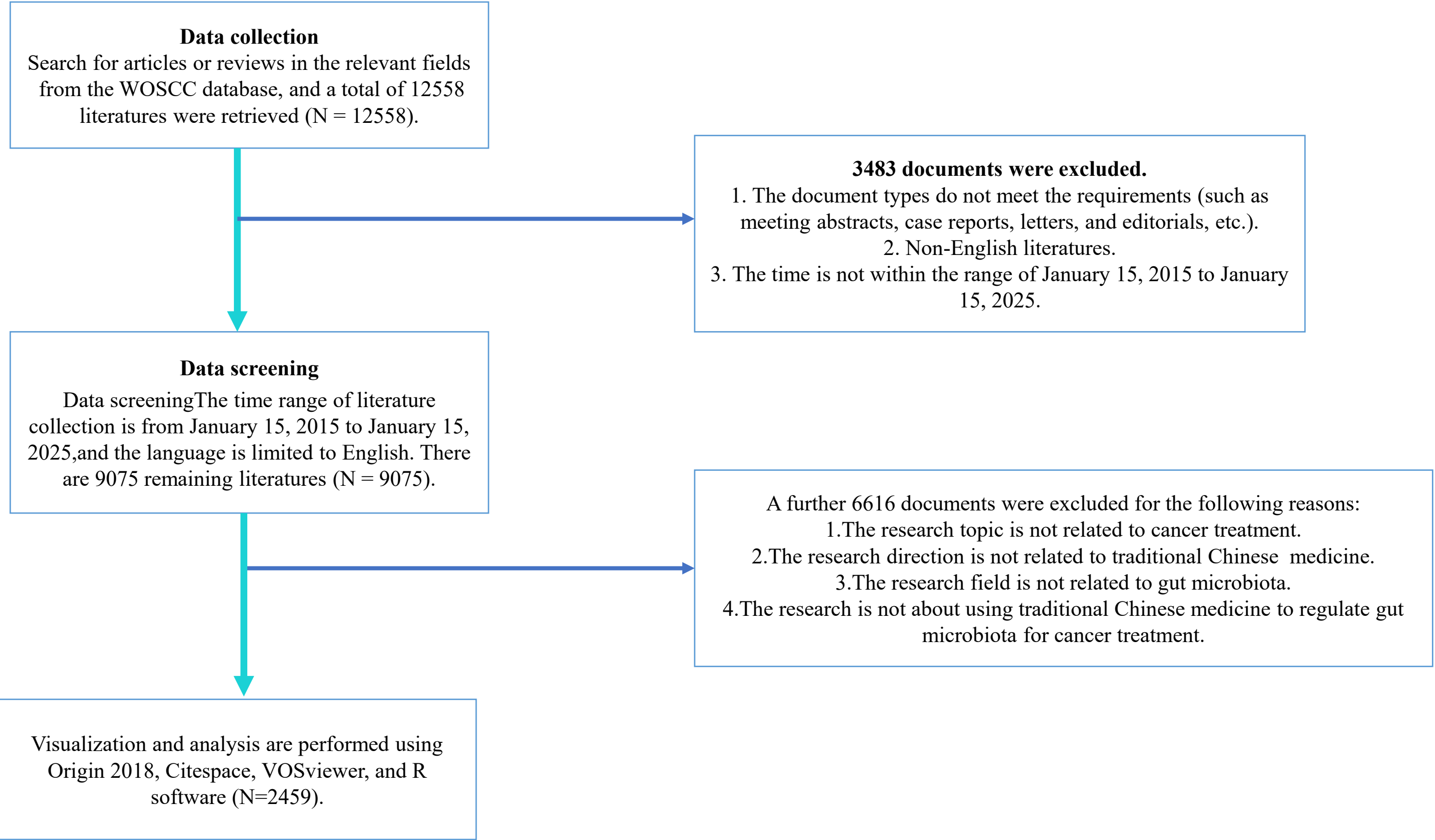

Supplement: Supplementary file 1 [file SupplementaryFile1.pdf]
