# Supplementary material for: Bibliometric analysis of traditional Chinese medicine in cancer treatment via immune system modulation (2015–2025)
Source: Front Immunol. 2025 May 8;16:1581885. doi: 10.3389/fimmu.2025.1581885 (PMC12095241; doi:10.3389/fimmu.2025.1581885)
Supplement: Supplementary file 2 [file SupplementaryFile2.docx]

| **References** | **Title** |
| --- | --- |
| Podolsky DK, 2002, NEW ENGL J MED, V347, P417, DOI | Inflammatory bowel disease |
| Baumgart DC, 2007, LANCET, V369, P1627, DOI 10.1016/S0140-  6736(07)60750-8, DOI | Inflammatory bowel disease: cause and immunobiology |
| Molodecky NA, 2012, GASTROENTEROLOGY, V142, P46, DOI | Increasing Incidence and Prevalence of the Inflammatory Bowel Diseases With Time, Based on Systematic Review |
| 10.1053/j.gastro.2011.10.001, DOI | Genetics and pathogenesis of inflammatory bowel disease |
| Neurath MF, 2014, NAT REV IMMUNOL, V14, P329, DOI 10.1038/nri3661, DOI | Cytokines in inflammatory bowel disease |
| Ordás I, 2012, LANCET, V380, P1606, DOI 10.1016/S0140-6736(12)60150-0, DOI | Ulcerative colitis |
| Jostins L, 2012, NATURE, V491, P119, DOI 10.1038/nature11582,DOI | Host–microbe interactions have shaped the genetic architecture of inflammatory bowel disease |
| Chassaing Benoit, 2014, CURR PROTOC IMMUNOL, V104, P0, DOI 10.1002/0471142735.im1525s104, DOI | Dextran sulfate sodium (DSS)-induced colitis in mice |
| de Souza HSP, 2016, NAT REV GASTRO HEPAT, V13, P13, DOI 10.1038/nrgastro.2015.186, 10.1038/nrgastro.2016.186, DOI | Immunopathogenesis of IBD: current state of the art,Steatohepatitis: PARP inhibition protective against alcoholic steatohepatitis and NASH |
| Ananthakrishnan AN, 2015, NAT REV GASTRO HEPAT, V12, P205, DOI 10.1038/nrgastro.2015.34, DOI | Epidemiology and risk factors for IBD |
| Kaplan GG, 2015, NAT REV GASTRO HEPAT, V12, P720, DOI 10.1038/nrgastro.2015.150, DOI | The global burden of IBD: from 2015 to 2025 |
| Ungaro R, 2017, LANCET, V389, P1756, DOI 10.1016/S0140 6736(16)32126-2, DOI | Ulcerative colitis |
| Ng SC, 2017, LANCET, V390, P2769, DOI 10.1016/S0140-6736(17)32448-0, DOI | Worldwide incidence and prevalence of inflammatory bowel disease in the 21st century: a systematic review of population-based studies |
| Kaplan GG, 2017, GASTROENTEROLOGY, V152, P313, DOI 10.1053/j.gastro.2016.10.020, DOI | Understanding and Preventing the Global Increase of Inflammatory Bowel Disease |
| Magro F, 2017, J CROHNS COLITIS, V11, P649, DOI 10.1093/ecco-jcc/jjx008, DOI | Third European Evidence-based Consensus on Diagnosis and Management of Ulcerative Colitis. Part 1: Definitions, Diagnosis, Extra-intestinal Manifestations, Pregnancy, Cancer Surveillance, Surgery, and Ileo-anal Pouch Disorders |
| Naganuma M, 2018, GASTROENTEROLOGY, V154, P935, DOI 10.1053/j.gastro.2017.11.024, DOI | Efficacy of Indigo Naturalis in a Multicenter Randomized Controlled Trial of Patients With Ulcerative Colitis |
| Gu PQ, 2017, INT IMMUNOPHARMACOL, V50, P152, DOI 10.1016/j.intimp.2017.06.022, DOI | Protective effects of paeoniflorin on TNBS-induced ulcerative colitis through inhibiting NF-kappaB pathway and apoptosis in mice |
| Wirtz S, 2017, NAT PROTOC, V12, P1295, DOI 10.1038/nprot.2017.044,DOI | Chemically induced mouse models of acute and chronic intestinal inflammation |
| Rubin DT, 2019, AM J GASTROENTEROL, V114, P384, DOI 10.14309/ajg.0000000000000152, DOI | ACG Clinical Guideline: Ulcerative Colitis in Adults |
| Nishida A, 2018, CLIN J GASTROENTEROL, V11, P1, DOI 10.1007/s12328 017-0813-5, DOI | Erratum to: Intestinal necrosis due to norovirus enteritis |
| Venegas DP, 2019, FRONT IMMUNOL, V10, P0, DOI 10.3389/fimmu.2019.00277, DOI | Short Chain Fatty Acids (SCFAs)-Mediated Gut Epithelial and Immune Regulation and Its Relevance for Inflammatory Bowel Diseases |
| Yao DB, 2019, INFLAMM BOWEL DIS, V25, P1595, DOI 10.1093/ibd/izz149, DOI | Inflammation and Inflammatory Cytokine Contribute to the Initiation and Development of Ulcerative Colitis and Its Associated Cancer |
| Tatiya-aphiradee Nitima, 2019, ...... LOGY AND PHARMACOLOGY, V30, P1, DOI | Immune response and inflammatory pathway of ulcerative colitis |
| Ramos GP, 2019, MAYO CLIN PROC, V94, P155, DOI 10.1016/j.mayocp.2018.09.013, DOI | Mechanisms of Disease: Inflammatory Bowel Diseases |
| Cui L, 2021, INT J BIOL MACROMOL, V166, P1035, DOI 10.1016/j.ijbiomac.2020.10.259, DOI | Scutellaria baicalensis Georgi polysaccharide ameliorates DSS-induced ulcerative colitis by improving intestinal barrier function and modulating gut microbiota |
