# Supplementary material for: Bibliometric analysis of traditional Chinese medicine in cancer treatment via immune system modulation (2015–2025)
Source: Front Immunol. 2025 May 8;16:1581885. doi: 10.3389/fimmu.2025.1581885 (PMC12095241; doi:10.3389/fimmu.2025.1581885)
Supplement: Supplementary file 3 [file SupplementaryFile3.docx]

**Cluster1(52)**

acid

anti-inflammatory

anticancer

antioxidant

antitumor

aqueous extract

bioactive compounds

chemical-composition

constituents

cyclophosphamide

cytotoxicity

extract

flavonoids

gene-expression

growth-performance

gut microbiota

health

identification

immune-response

immune-responses

immune-system

immunity

immunomodulation

immunomodulatory

immunosuppression

in-vitro

innate

innate immunity

l.

leaves

liver

medicinal mushrooms

medicinal-plants

metabolism

microbiota

oxidative stress

panax-ginseng

performance

pharmacokinetics

pharmacology

phytochemicals

phytochemistry

polyphenols

polysaccharide

polysaccharides

protein

purification

saponins

stress

structural-characterization

supplementation

toxicity

**Cluster2(51)**

antitumor immunity

blockade

breast cancer

cancer immunotherapy

carcinoma

cell lung-cancer

cell-death

chemotherapy

chinese medicine

combination

delivery

dendritic cells

double-blind

doxorubicin

efficacy

expression

gastric cancer

gastric-cancer

herbal medicine

immune checkpoint inhibitors

immunotherapy

lung cancer

management

melanoma

meta-analysis

metastasis

nanoparticles

natural products

nivolumab

nk cells

non-small cell lung cancer

pd-1

pd-l1

polarization

prevention

prognosis

progression

promotes

quality-of-life

regulatory t-cells

resistance

risk

safety

stat3

statistics

suppressor-cells

survival

t-cells

therapy

tumor microenvironment

tumor-associated macrophages

**Cluster3 (38)**

activation

acute lung injury

alpha

anti-inflammation

atopic dermatitis

colitis

cytokine

differentiation

disease

gene

infection

inflammation

inflammatory bowel disease

inhibition

injury

kappa-b

lipopolysaccharide

lymphocytes

macrophage

mechanisms

mice

model

modulation

mouse model

nf-kappa b

nitric-oxide

nlrp3 inflammasome

pathogenesis

pathways

rats

receptor

rheumatoid arthritis

sepsis

suppression

system

tnf-alpha

tumor-necrosis-factor

ulcerative colitis

**Cluster4(26)**

angiogenesis

apoptosis

autophagy

breast-cancer cells

cancer cells

cell-cycle arrest

colon cancer

colorectal cancer

curcumin

cycle arrest

down-regulation

epithelial-mesenchymal transition

growth

hepatocellular carcinoma

in-vivo

induced apoptosis

invasion

lung-cancer

mechanism

migration

molecular-mechanisms

natural-products

pathway

proliferation

signaling pathway

up-regulation

**Cluster5(4)**

induction

molecular docking

network pharmacology

quercetin
